# Childhood trauma, posttraumatic stress disorder symptoms, early maladaptive schemas, and schema modes: a comparison of individuals with obesity and normal weight controls

**DOI:** 10.1186/s12888-022-04169-7

**Published:** 2022-07-30

**Authors:** Dean Spirou, Jayanthi Raman, Ramy H. Bishay, Golo Ahlenstiel, Evelyn Smith

**Affiliations:** 1 Discipline of Clinical Psychology, Graduate School of Health, University of Technology Sydney, Sydney, NSW Australia; 2 Blacktown Metabolic and Weight Loss Program, Department of Endocrinology and Diabetes, Blacktown Hospital, Blacktown, Sydney, NSW Australia; 3 School of Medicine, Western Sydney University, Sydney, NSW Australia; 4 School of Psychological Sciences, University of Newcastle, Newcastle, NSW Australia; 5 Storr Liver Centre, Westmead Millennium Institute, Westmead Hospital, Sydney, NSW Australia; 6 Department of Gastroenterology and Hepatology, WSLHD, Blacktown and Mount Druitt Hospitals, Sydney, NSW Australia; 7 School of Psychology, Western Sydney University, Sydney, NSW Australia; 8 Translational Health Research Institute, Western Sydney University, Sydney, NSW Australia

**Keywords:** Obesity, Early maladaptive schemas, Schema modes, Schema therapy, Childhood trauma, Posttraumatic stress disorder

## Abstract

**Background:**

Previous research on the psychological mechanisms of obesity has primarily focused on acute psychopathology. However, there is limited literature on the role of more complex and entrenched psychological processes in weight management. The current study aimed to expand previous research by examining more enduring psychological constructs, including early maladaptive schemas (EMS), schemas modes, and trauma.

**Methods:**

Participants (*N* = 125) comprised adults with normal weight (*n* = 40) and obesity (*n* = 85) from community and clinical settings in Australia. Eligible participants completed a series of self-report questionnaires via Research Electronic Data Capture (REDCap). Two, separate, one-way multivariate analysis of variance (MANOVA) were conducted to examine group differences on the outcome variables.

**Results:**

Findings indicated a significant effect of group on EMS and schema modes, V = .51, *F*(32, 92) = 2.97, *p* < .001, partial η^2^ = .51. Follow-up univariate tests revealed that individuals with obesity endorsed significantly more maladaptive schemas and schema modes and significantly less healthy schema modes than individuals with normal weight. In addition, results demonstrated a significant effect of group on childhood trauma and posttraumatic stress disorder (PTSD) symptoms, V = .19, *F*(6, 118) = 4.70, *p* < .001, partial η^2^ = .19. Subsequent univariate tests and chi-square analyses indicated that individuals with obesity reported significantly more childhood trauma as well as significantly more PTSD symptoms within the last month than normal weight individuals.

**Conclusion:**

This was the first study to compare EMS and schema modes in treatment-seeking individuals with obesity and normal weight controls using the short form version 3 of the Young Schema Questionnaire and revised, 118-item, Schema Mode Inventory. Overall, findings revealed that individuals with obesity experience more complex and enduring psychological difficulties than normal weight individuals. Increased assessment and targeted treatment of these underlying mental health concerns may contribute to a more holistic conceptualisation of obesity and could improve the long-term success of weight management.

**Supplementary Information:**

The online version contains supplementary material available at 10.1186/s12888-022-04169-7.

## Introduction

Preventing weight regain after weight loss intervention remains the single greatest challenge for individuals with obesity [1–3]. Successful weight maintenance requires long-term modification of diet and physical activity as well ongoing support [1–4]. Psychological factors may impede adherence to diet and physical activity and may hinder long-term weight loss maintenance [1, 4–6]. Research on the psychological mechanisms of obesity has predominantly focused on temporary or acute difficulties (e.g., depression, anxiety, binge eating), with limited attention on more complex and entrenched psychological processes. Recently, however, researchers have begun examining more enduring psychological constructs such as early maladaptive schemas (EMS), schemas modes, and trauma.

EMS are central to schema therapy, an integrated psychotherapy incorporating and extending elements of cognitive-behavioural therapy, Gestalt therapy, psychodynamic therapy, and attachment theory [7]. EMS refer to pervasive and enduring life patterns regarding oneself and one’s relationship with others, and consist of memories, cognitions, physiological sensations, and emotions [7, 8]. EMS develop during childhood or adolescence and strengthen over time [7]. They are theorised to be a result of unmet core emotional needs in childhood (e.g., secure attachment to others, freedom to express valid needs and emotions), primarily due to the interplay between adverse childhood experiences (e.g., traumatisation or victimisation) and emotional temperament [7]. Young et al. [7] proposed 18 EMS that are categorised into five domains: Disconnection and Rejection, Impaired Autonomy and Performance, Impaired Limits, Other-Directedness, and Overvigilance and Inhibition (see Additional File 1 for a description of EMS) [7]. These schemas are initially adaptive coping responses to a child’s environment but become maladaptive over time and may elicit significant impairment [7, 8].

Another key concept in schema therapy is the notion of schema modes. Schema modes are moment-to-moment coping responses and emotional states associated with one or several EMS (see Additional File 2 for a description of schema modes) [7]. Schema modes may be adaptive or maladaptive and are often triggered by contextual circumstances [7]. Arntz and Jacob [8] referred to EMS as being conceptually similar to traits, with schema modes depicting the states associated with those schemas. Four categories of modes have been proposed, including Child modes, Dysfunctional Parent modes, Dysfunctional Coping modes, and Healthy modes [7, 8]. Child modes are considered universal and innate and resemble the notion of the “inner child” [7, 8]. Dysfunctional Parent modes represent the individuals’ internalisation of dysfunctional parenting such as placing excessive pressure on oneself to meet high standards (e.g., critical or demanding voice) [7, 8]. Dysfunctional Coping modes reflect an individual’s unhelpful coping strategies such as avoidance, overcompensation, or surrendering [7, 8]. Finally, the Healthy modes represent one’s ability to realistically view themselves and life, and feel loved, connected, and joyful [7, 8].

Furthermore, early childhood experiences are a critical component in schema therapy. Young et al. [7] asserted that adverse childhood experiences (e.g., toxic frustration of needs) are the primary origin for EMS. Supporting this notion, Pilkington et al. [9] found small to large associations between childhood adversity and EMS in adulthood, with the strongest link to a history of childhood emotional abuse. In addition, previous research has demonstrated a positive association between traumatic experiences (e.g., sexual abuse, physical abuse, peer bullying, interpersonal violence) and obesity [10–12] as well as posttraumatic stress disorder (PTSD) and obesity [12], with significantly greater impairment to self-esteem, psychosocial functioning, body image, physical health, and eating pathology among those with a lifetime history of PTSD [13]. Research has also demonstrated a link between traumatic experiences and eating disorders such as binge eating disorder (BED) [12, 14]. Together, these findings emphasise the heightened risk of developing maladaptive schemas, eating disorders, and obesity for those with a history of trauma.

In recent years, the schema model has received increasing attention in the field of obesity. Thus far, three studies have utilised the Young Schema Questionnaire, Short Form, Version 2 (YSQ-S2) to compare EMS in individuals with overweight and obesity and normal weight controls [15–17]. First, Anderson et al. [15] found that individuals with obesity had significantly higher scores on the Defectiveness/Shame, Social Isolation/Alienation, and Failure schemas relative to normal weight controls. In addition, Basile et al. [16] reported that the Abandonment, Insufficient Self-Control, Subjugation, and Dependency schemas were significantly higher among the overweight and obesity group compared to the normal weight group. In contrast, however, da Luz et al. [17] found that individuals with obesity only had significantly higher scores on the Insufficient Self-Control schema compared to normal weight individuals, but this effect became non-significant after controlling for mental health. One explanation for this discrepancy is that da Luz et al. [17] employed stricter exclusion criteria regarding age, education, and cognitive ability, which limited the variability in their sample. In addition, more than half (54%) of their sample were receiving psychiatric medication at the time of the study, which may have attenuated the activation or presence of EMS, though further research is required to verify this claim. Moreover, one study has investigated differences in schema modes across weight classes using the 124-item Schema Mode Inventory [16]. Basile et al. [16] found that the Vulnerable Child, Impulsive Child, and Detached Protector modes were significantly higher among the overweight and obesity group, while the Happy Child and Healthy Adult modes were significantly higher among the normal weight group.

To date, no study has utilised the Young Schema Questionnaire, Short Form, Version 3 (YSQ-S3) to compare EMS in individuals with obesity and normal weight controls. In addition, previous studies using the YSQ-S2 have found inconsistent results regarding group differences. Further, the only study examining schema modes in obesity combined overweight and obesity participants from the community and utilised an earlier iteration of the Schema Mode Inventory, containing 124-items [16]. To our knowledge, no study has used the refined, 118-item, Short Schema Mode Inventory (SMI) to examine schema modes in treatment-seeking individuals with obesity and normal weight controls. Similarly, previous research on trauma and obesity has primarily focused on individuals in the community, with less emphasis on comparing differences in individuals with obesity attending tertiary services and normal weight controls. Investigating these relationships will contribute to the growing literature on trauma and schema modes in obesity and will contribute to clarifying inconsistent findings in the literature regarding EMS.

The goal of the current study was to examine whether there were significant differences in EMS and schema modes among treatment-seeking individuals with obesity and normal weight controls. This study extends previous research by comparing a normal weight control group to individuals with obesity attending a tertiary weight intervention service, and by utilising the latest iteration of the schema questionnaires, namely the YSQ-S3 and revised SMI. In addition, we aimed to investigate whether individuals with obesity and normal weight significantly differed on childhood trauma and PTSD symptoms. In light of previous research, we hypothesised that the obesity group would have significantly higher scores on the Abandonment, Defectiveness/Shame, Social Isolation/Alienation, Dependence/Incompetence, Failure, Insufficient Self-Control, and Subjugation schemas relative to normal weight controls. Given the existing literature on trauma and obesity, we also hypothesised that individuals with obesity would have significantly higher scores on the Mistrust/Abuse and Emotional Deprivation schemas compared to normal weight individuals. In addition, we predicted that individuals with obesity would have significantly higher scores on the Vulnerable Child, Impulsive Child, and Detached Protector schema modes, but significantly lower scores on the Happy Child and Healthy Adult modes compared to normal weight controls. Further, we hypothesised that the obesity group would have significantly higher scores on childhood trauma and PTSD symptoms compared to the normal weight group.

## Method

### Participants

Participants (*N* = 163) comprised adults with normal weight (*n* = 58) and obesity (*n* = 105) from Australia. This data overlaps with another study submitted for publication by the same authors, though focuses on distinct research questions and statistical analyses. Normal weight participants were recruited via community advertisements and social media. Participants with normal weight were eligible if aged 18 years or older and BMI was between 18.5 to 24.9 kg/m^2^. Participants with obesity were recruited from the Blacktown Metabolic and Weight Loss Program (BMWLP) at Blacktown Hospital, prior to commencing treatment. The BMWLP is a tertiary level service that provides multidisciplinary weight intervention for adults with obesity, including the option for bariatric surgery for eligible patients. To be eligible for enrolment in the BMWLP, participants required a BMI $$\ge$$ 35 kg/m^2^ with type 2 diabetes or a BMI $$\ge$$ 40 kg/m^2^ with at least two obesity-related complications (e.g., hypertension, hyperlipidaemia, sleep apnoea, fatty liver disease, cardiac disease, stroke disease, or joint disease). Recruitment occurred across a 12-month period. All participants were informed about the research aims. Participants in either group were excluded if they had a cognitive or speech impairment, neurological disorder, intellectual disability, head injury, or mental health condition which interfered with their ability to complete or understand the requirements of the study. Eligible participants completed a series of self-report questionnaires via Research Electronic Data Capture (REDCap), a secure web-based software platform [18, 19]. The questionnaires took approximately 45–60 min to complete. Community participants were offered a $15 gift voucher as reimbursement. This study was approved by the Western Sydney Local Health District (5450 – 2019/ETH01915) and was ratified by the University of Technology Sydney (ETH20-5545; ETH20-6063).

Of the 163 participants, 37 were excluded because of missing data. One significant outlier was also excluded. The final sample (*N* = 125) included males (32.8%) and females (67.2%) that ranged in age from 21 to 79 years (*M* = 41.33, *SD* = 13.98) and ranged in BMI from 19.1 kg/m^2^ to 75.4 kg/m^2^ (*M* = 42.04, *SD* = 15.77). Of these 125 participants, 40 (32%) were in the normal weight group and 85 (68%) were in the obesity group. Table 1 presents demographic characteristics of the final sample.

**Table 1:** Demographic Characteristics of the Sample

Variable	Normal weight (*n* = 40)	Obesity (*n* = 85)	Total sample (*N* = 125)
Age ^a^	31.78 (10.04)	45.82 (13.34)	41.33 (13.98)
BMI ^a^	22.59 (1.84)	51.19 (10.03)	42.04 (15.77)
Sex ^b^			
Male	6 (15.0%)	35 (41.2%)	41 (32.8%)
Female	34 (85.0%)	50 (58.8%)	84 (67.2%)
Employment status ^b^			
Unemployed	––	51 (60.0%)	51 (40.8%)
Employed	33 (82.5%)	21 (24.7%)	54 (43.2%)
Studying	7 (17.5%)	2 (2.4%)	9 (7.2%)
Carer	––	6 (7.1%)	6 (4.8%)
Retired	––	5 (5.8%)	5 (4.0%)
Highest education level ^b^			
Less than Year 10	––	12 (14.1%)	12 (9.6%)
High school (Year 10)	––	20 (23.5%)	20 (16.0%)
High school (Year 11)	––	4 (4.7%)	4 (3.2%)
High school (Year 12)	1 (2.5%)	13 (15.3%)	14 (11.2%)
College/TAFE ^b^	5 (12.5%)	28 (32.9%)	33 (26.4%)
Bachelor’s degree	15 (37.5%)	7 (8.2%)	22 (17.6%)
Master’s degree	16 (40.0%)	1 (1.2%)	17 (13.6%)
Doctorate	3 (7.5%)	––	3 (2.4%)

*Note.* Missing values not included. *BMI* body mass index

^a^ *M* (*SD)*

^b^ *n* (*%*)

### Measures

#### Demographics and Eligibility

Sociodemographic information (i.e., sex, age, employment status, education) was collected through a general self-report questionnaire. Mental and physical health information was also collected to determine participant eligibility.

#### Anthropometrics

Specialised wheelchair scales were used to measure participant height and weight for the obesity group. Self-reported height and weight was used for the normal weight group due to changes in recruitment procedures associated with COVID-19. BMI was computed by dividing weight in kilograms by height in metres squared (kg/m^2^).

#### Young Schema Questionnaire – Short Form, Version 3 (YSQ-S3)

The YSQ-S3 is a 90-item self-report measure of EMS [20]. The YSQ-S3 comprises 18 scales (schemas) with 5 items in each scale. Participants rate a series of statements according to how accurately it reflects them over the past year. Items are rated on a 6-point Likert scale ranging from 1 (*completely untrue of me*) to 6 (*describes me perfectly*). A total score for each scale is derived by summing the scale items. Higher scores indicate greater endorsement of that schema. The YSQ-S3 has demonstrated good internal consistency in clinical and non-clinical samples across several languages (e.g., [21–24]).

#### Short Schema Mode Inventory (SMI)

The SMI is a 118-item self-report measure of moment-to-moment coping responses and cognitive and emotional states [25]. The SMI assesses 14 scales (schema modes) with 4 to 10 items in each scale. Items are rated on a 6-point Likert scale ranging from 1 (*never or almost never*) to 6 (*all of the time*). The score for each scale is derived by calculating the scale mean. Higher scores indicate greater endorsement of that schema mode. The SMI has demonstrated acceptable internal consistency and test–retest reliability [25].

#### Childhood Trauma Questionnaire – Short Form (CTQ-SF)

The CTQ-SF is a 28-item self-report screening measure of retrospective childhood abuse and neglect [26]. The CTQ-SF comprises five clinical scales: Emotional Abuse, Physical Abuse, Sexual Abuse, Emotional Neglect, and Physical Neglect. Each scale contains five items. Items are rated on a 5-point Likert scale ranging from 1 (*never true*) to 5 (*very often true*). The total score for each scale ranges from 5 to 25 with higher scores suggesting greater exposure to maltreatment. Two main approaches have been applied to interpreting the CTQ-SF. First, the total score for each scale can be classified according to four levels of maltreatment: None (or Minimal), Low (to Moderate), Moderate (to Severe), and Severe (to Extreme) [26]. Second, a dichotomous cut-off score can be used to differentiate the presence or absence of maltreatment [27]. Using the dichotomous approach, scores $\ge$ 10 for Emotional Abuse, 8 for Physical Abuse, 8 for Sexual Abuse, 15 for Emotional Neglect, and 8 for Physical Neglect indicate the presence of maltreatment [27]. The CTQ-SF also contains a 3-item Minimisation/Denial scale assessing possible underreporting of maltreatment. On this scale, items rated 5 (*very often true*) were coded as 1 (*possible underreporting*), while items rated 1 (*never true*) through 4 (*often true*) were coded as 0 (*no underreporting*). The validity and reliability of the CTQ-SF has been well-supported across both clinical and non-referred samples [26].

#### PTSD Checklist for DSM-5 (PCL-5)

The PCL-5 is a 20-item self-report measure of PTSD symptoms within the past month [28]. Items are rated on a 5-point Likert scale ranging from 0 (*not at all*) to 4 (*extremely*). A total score ranging from 0 to 80 is calculated by summing the items. Higher scores indicate a greater frequency of PTSD symptoms. A provisional PTSD diagnosis is indicated using a cut-off score of 31 to 33. In the current study, participants scoring above 31 were coded as 1 (*provisional diagnosis*), while participants scoring below 31 were coded as 0 (*no diagnosis*). Each item also corresponds with a symptom of PTSD in the Diagnostic and Statistical Manual of Mental Disorders-fifth edition (DSM-5) [29]. Corresponding items scored 2 (*moderately*) or higher indicate a DSM-5 symptom has been endorsed. The PCL-5 has demonstrated high internal consistency, test–retest reliability, and convergent and discriminant validity [30, 31].

### Data Analyses

All analyses were carried out using SPSS Version 28.0. Initially, the data was inspected for outliers and missing values, and assumption testing was performed. Missing data was managed through listwise deletion. Two, separate, one-way multivariate analysis of variance (MANOVA) were conducted to examine group differences on the outcome variables. The choice to conduct two separate MANOVA was based on the theoretical and empirical relationship between these variables, and the research aims. It was also anticipated that two separate MANOVA would likely yield the most clinically meaningful interpretations given the paucity of research in this area. Further, fitting separate models has been recommended when there are reasonable theoretical grounds for separating outcome measures [32]. First, a one-way MANOVA was performed to explore group differences on EMS and schema modes. Second, a one-way MANOVA was conducted to examine group differences on childhood trauma and PTSD symptoms. In addition, chi-square (χ^2^) analyses were performed to investigate whether groups differed on childhood trauma and provisional PTSD diagnosis using the dichotomous scoring method on the CTQ-SF and PCL-5, respectively. Pillai’s trace (V) was used to interpret the MANOVA results. Pillai’s trace has been recommended as the statistic of choice because of its robustness to model violations [33–35]. Following a significant MANOVA, separate univariate tests were conducted to examine group differences on the outcome variables. Partial eta-squared (η^2^) was used as the effect size (.01 = small, .06 = medium, and .14 = large). Bonferroni correction was applied to circumvent inflation of the type 1 error rate due to multiple comparisons. The alpha level for the first and second MANOVA were adjusted to .002 and .008, respectively.

## Results

### Data Screening

Data from the total sample (*N* = 125) was utilised to examine group differences on the outcome variables. All observations were independent, measured on a ratio scale, and randomly sampled from the populations of interest. There were 33 (38.8%) participants in the obesity group and 10 (25%) participants in the normal weight group with scores on the CTQ-SF that suggested possible underreporting of maltreatment (i.e., false negatives). These participants were retained in the analyses. There were no concerns with multicollinearity and there was sufficient observed power (> .80) for all significant effects. Multivariate normality and homogeneity of covariance matrices were partially satisfactory. These model assumptions, however, are susceptible to deviations in large samples [32]. Nevertheless, the central limit theorem asserts that sampling distribution will be normally distributed in large samples [32, 35].

### Comparing EMS and Schema Modes Across Groups

Using Pillai’s trace, there was a significant effect of group on EMS and schema modes, V = .51, *F*(32, 92) = 2.97, *p* < .001, partial η^2^ = .51. As hypothesised, separate univariate tests on the outcome variables revealed that individuals with obesity had significantly higher scores on the Abandonment (*p* < .001), Mistrust/Abuse (*p* < .001), Emotional Deprivation (*p* < .001), Defectiveness/Shame (*p* < .001), Social Isolation/Alienation (*p* < .001), Dependence/Incompetence (*p* < .001), Failure (*p* < .001), Insufficient Self-Control (*p* < .001), and Subjugation (*p* < .001) schemas compared to normal weight controls. Unexpectedly, individuals with obesity also had significantly higher scores on the Vulnerability to Harm and Illness (*p* < .001), Self-Sacrifice (*p* = .002), Negativity/Pessimism (*p* < .001), and Emotional Inhibition (*p* < .001) schemas. Furthermore, as predicted, individuals with obesity demonstrated significantly higher scores on the Vulnerable Child (*p* < .001), Impulsive Child (*p* < .001), and Detached Protector (*p* < .001) schema modes as well as significantly lower scores on the Happy Child (*p* < .001) and Healthy Adult (*p* < .001) modes compared to normal weight controls. Unexpectedly, individuals with obesity also exhibited significantly higher scores on the Angry Child (*p* = .002), Undisciplined Child (*p* < .001), Compliant Surrender (*p* < .001), and Punitive Parent (*p* < .001) modes. Table 2 presents the means, standard deviations, and univariate outcomes for EMS and schema modes.

**Table 2:** Means, Standard Deviations, and One-Way ANOVA Results for Early Maladaptive Schemas and Schema Modes

Variable	Normal weight		Obesity		*F*(1,123)	*p*	Partial η^2^
	*M*	*SD*	*M*	*SD*			
Early maladaptive schemas							
Abandonment	8.75	3.71	13.34	6.81	15.92	< .001 ^*^	.12
Mistrust/abuse	8.47	4.35	13.92	6.90	20.92	< .001 ^*^	.15
Emotional deprivation	7.80	4.64	13.71	6.79	24.79	< .001 ^*^	.17
Defectiveness/shame	7.03	2.95	13.29	7.52	25.87	< .001 ^*^	.17
Social isolation/alienation	8.85	4.59	14.61	7.63	19.43	< .001 ^*^	.14
Dependence/incompetence	7.35	2.82	11.65	4.99	25.69	< .001 ^*^	.17
Vulnerability to harm and illness	8.88	4.29	12.76	6.67	11.37	< .001 ^*^	.09
Enmeshment	7.28	2.98	9.71	5.11	7.78	.006	.06
Failure	8.20	3.47	13.36	6.94	19.74	< .001 ^*^	.14
Entitlement/grandiosity	9.63	4.25	11.14	5.02	2.72	.101	.02
Insufficient self-control	9.60	3.91	13.65	5.96	15.32	< .001 ^*^	.11
Subjugation	8.48	3.95	11.95	5.38	13.30	< .001 ^*^	.10
Self-sacrifice	14.02	5.30	17.71	6.36	10.12	.002 ^*^	.08
Approval-seeking	10.85	3.22	11.91	5.82	1.15	.286	.01
Negativity/pessimism	9.90	4.76	14.09	6.91	12.01	< .001 ^*^	.09
Emotional inhibition	9.33	3.70	13.13	5.99	13.65	< .001 ^*^	.10
Unrelenting standards	14.50	5.64	15.11	5.90	0.30	.588	.00
Punitiveness	8.90	3.42	12.14	6.22	9.49	.003	.07
Schema modes							
Vulnerable child	1.90	0.66	3.11	1.26	32.60	< .001 ^*^	.21
Angry child	1.94	0.63	2.44	0.91	10.27	.002 ^*^	.08
Enraged child	1.27	0.38	1.63	0.85	6.53	.012	.05
Impulsive child	1.84	0.64	2.40	0.89	12.93	< .001 ^*^	.10
Undisciplined child	2.27	0.66	3.21	0.97	30.83	< .001 ^*^	.20
Happy child	4.56	0.69	3.47	1.06	35.34	< .001 ^*^	.22
Compliant surrender	2.58	0.71	3.20	0.92	14.02	< .001 ^*^	.10
Detached protector	1.75	0.63	2.64	1.04	24.86	< .001 ^*^	.17
Detached self-soother	2.52	1.02	2.98	0.95	6.04	.015	.05
Self-aggrandiser	2.44	0.56	2.27	0.66	2.00	.160	.02
Bully/attack	1.74	0.42	1.99	0.71	4.04	.047	.03
Punitive parent	1.68	0.58	2.41	0.97	19.56	< .001 ^*^	.14
Demanding parent	3.23	0.89	3.27	1.02	0.05	.817	.00
Healthy adult	4.84	0.49	4.04	0.82	31.90	< .001 ^*^	.21

*Note. N* = 125 (*n* = 40 for the normal weight group, *n* = 85 for the obesity group). *ANOVA* analysis of variance

^*^ *p* < .002 (Bonferroni correction)

### Comparing Childhood Trauma and PTSD Symptoms Across Groups

Using Pillai’s trace, there was a significant effect of group on childhood trauma and PTSD symptoms, V = .19, *F*(6, 118) = 4.70, *p* < .001, partial η^2^ = .19. As hypothesised, separate univariate tests on the outcome variables revealed that individuals with obesity had significantly higher scores on Emotional Abuse (*p* < .001), Physical Abuse (*p* < .001), Sexual Abuse (*p* = .005), Emotional Neglect (*p* = .004), Physical Neglect (*p* < .001), and PTSD symptoms (*p* < .001) compared to normal weight controls. Table 3 presents the means, standard deviations, and univariate outcomes for childhood trauma and PTSD symptoms.

**Table 3:** Means, Standard Deviations, and One-Way ANOVA Results for Trauma Variables

Variable	Normal weight		Obesity		*F*(1,123)	*p*	Partial η^2^
	*M*	*SD*	*M*	*SD*			
Emotional abuse	8.50	3.61	13.04	6.56	16.68	< .001 ^*^	.12
Physical abuse	6.18	1.48	9.78	5.36	17.35	< .001 ^*^	.12
Sexual abuse	5.43	1.82	8.29	6.14	8.35	.005 ^*^	.06
Emotional neglect	9.03	3.95	12.15	6.12	8.71	.004 ^*^	.07
Physical neglect	6.18	1.74	9.24	4.42	17.81	< .001 ^*^	.13
PTSD symptoms	12.77	14.27	26.26	19.38	15.41	< .001 ^*^	.11

*Note. N* = 125 (*n* = 40 for the normal weight group, *n* = 85 for the obesity group). *ANOVA* analysis of variance, *PTSD* posttraumatic stress disorder

^*^ *p* < .008 (Bonferroni correction)

Using the dichotomous scoring method on the CTQ-SF and PCL-5, chi-square analyses revealed that individuals with obesity reported a significantly higher proportion of Emotional Abuse, χ^2^ (1, *N* = 125) = 6.18, *p* = .013, Physical Abuse, χ^2^ (1, *N* = 125) = 8.16, *p* = .004, Sexual Abuse, χ^2^ (1, *N* = 125) = 8.91, *p* = .003, Emotional Neglect, χ^2^ (1, *N* = 125) = 8.78, *p* = .003, Physical Neglect χ^2^ (1, *N* = 125) = 14.93, *p* < .001, and provisional PTSD diagnosis, χ^2^ (1, *N* = 125) = 6.59, *p* = .010, compared to normal weight individuals. Table 4 presents frequency statistics for the presence of childhood trauma and provisional PTSD diagnosis using the dichotomous method on the CTQ-SF and PCL-5, respectively.

**Table 4:** Presence of Childhood Trauma and Provisional PTSD Diagnosis on the CTQ-SF and PCL-5 Using the Dichotomous Scoring Method

Variable	Normal weight		Obesity		χ^2^		
	*n*	%	*n*	%	Value	*df*	*p*
Emotional abuse	14	35.0	50	58.8	6.18	1	.013
Physical abuse	9	22.5	42	49.4	8.16	1	.004
Sexual abuse	2	5.0	24	28.2	8.91	1	.003
Emotional neglect	3	7.5	27	31.8	8.78	1	.003
Physical neglect	7	17.5	46	54.1	14.93	1	< .001
Provisional PTSD diagnosis	6	15.0	32	37.6	6.59	1	.010

*Note. N* = 125 (*n* = 40 for the normal weight group, *n* = 85 for the obesity group). *PTSD* posttraumatic stress disorder, *PCL-5* PTSD checklist for DSM-5, *CTQ-SF* childhood trauma questionnaire – short form

## Discussion

The current study was the first to compare early maladaptive schemas (EMS) and schema modes using the YSQ-S3 and revised SMI, respectively, in normal weight controls and individuals with obesity attending a tertiary weight intervention service. This study also investigated differences in childhood trauma and PTSD symptoms among treatment-seeking individuals with obesity and normal weight controls.

First, consistent with our hypotheses and previous research [15, 16], individuals with obesity endorsed significantly higher scores on the Abandonment, Mistrust/Abuse, Emotional Deprivation, Defectiveness/Shame, Social Isolation/Alienation, Dependence/Incompetence, Failure, Insufficient Self-Control, and Subjugation schemas compared to normal weight controls. Unexpectedly, individuals with obesity also demonstrated significantly higher scores on the Vulnerability to Harm and Illness, Self-Sacrifice, Negativity/Pessimism, and Emotional Inhibition schemas. Notably, our findings revealed that all schemas in the Disconnection and Rejection domain were significantly higher in the obesity group, with medium to large effect sizes. The Disconnection and Rejection domain primarily reflects difficulties with developing secure attachments to others [7, 8]. Previous research has demonstrated that elevations in this schema domain is related to eating disorders and predicts food addiction symptoms among women with overweight and obesity [36, 37].

Specifically, these results suggested that compared to normal weight individuals, those with obesity may perceive their important relationships with others as more unstable (i.e., Abandonment) or may believe that others are more likely to abuse and treat them poorly (i.e., Mistrust/Abuse) and that their emotional needs will remain unsatisfied (i.e., Emotional Deprivation) [7, 8]. They may also experience more shame or perceive themselves as more defective (i.e., Defectiveness/Shame) or have a stronger sense of not belonging to communities and social groups due to feeling dissimilar (i.e., Social Isolation/Alienation) [7, 8]. In addition, they may believe they are less capable of independently managing daily responsibilities (i.e., Dependence/Incompetence), that catastrophic illness and injury (e.g., heart attack) is unpreventable and imminent (i.e., Vulnerability to Harm and Illness), and that they will inevitably fail and be less successful than peers (i.e., Failure) [7, 8]. Furthermore, they may experience greater difficulties with self-control (i.e., Insufficient Self-Control), may be more likely to surrender control to others (i.e., Subjugation), or more likely to prioritise the needs of others at the expense of their own (i.e., Self-Sacrifice) [7, 8]. Moreover, they may exhibit a greater preoccupation with negative aspects of life (i.e., Negativity/Pessimism) as well as a greater inhibition of emotion due to the belief that emotions are unnecessary or unpleasant to display (i.e., Emotional Inhibition) [7, 8].

Second, consistent with our predictions and previous research [16], individuals with obesity demonstrated significantly higher scores on the Vulnerable Child, Impulsive Child, and Detached Protector modes as well as significantly lower scores on the Happy Child and Healthy Adult modes relative to normal weight individuals. Unexpectedly, individuals with obesity also displayed significantly higher scores on the Angry Child, Undisciplined Child, Compliant Surrender, and Punitive Parent modes. Specifically, these findings suggested that individuals with obesity experience a higher degree of sadness and desperation (i.e., Vulnerable Child) and may be more likely to act in anger and frustration due to unmet needs (i.e., Angry Child) or to behave in impulsive and uncontrolled ways to satisfy their needs (i.e., Undisciplined and Impulsive Child), but not in a more aggressive or violent manner than normal weight individuals (i.e., Enraged Child) [7, 8]. In addition, individuals with obesity more frequently experience a harsh, unforgiving, and critical internalised parent/caregiver voice (i.e., Punitive Parent) but place a degree of pressure on themselves to meet high standards that is similar to normal weight individuals (i.e., Demanding Parent) [7, 8]. Further, individuals with obesity adopt a more passive, submissive, and compliant coping method (i.e., Compliant Surrender) as well as a more avoidant, emotionally withdrawn, and detached coping style (i.e., Detached Protector). Moreover, normal weight individuals demonstrate less activated schemas, more satisfied core emotional needs, and more appropriate adult functioning than individuals with obesity (i.e., Happy Child, Healthy Adult) [7, 8].

Third, as hypothesised, individuals with obesity demonstrated significantly higher scores on Emotional Abuse, Physical Abuse, Sexual Abuse, Emotional Neglect, Physical Neglect, and PTSD symptoms compared to normal weight individuals. Significant differences between groups were also observed on childhood trauma and provisional PTSD diagnosis using the dichotomous scoring methods on the CTQ-SF and PCL-5. Specifically, 58.8% of individuals with obesity reported the presence of Emotional Abuse, 49.4% reported the presence of Physical Abuse, 28.2% reported the presence of Sexual Abuse, 31.8% reported the presence of Emotional Neglect, 54.1% reported the presence of Physical Neglect, and 37.6% reported PTSD symptoms within the last month that were above the cut-off criteria to suggest a provisional diagnosis of PTSD. These results support previous studies that found an increased probability of developing obesity for those with childhood traumatic experiences and PTSD [10–12, 38]. Similarly, these results are comparable with Walsh et al. [13], who found that one-third of their pre-bariatric surgery sample endorsed a history of physical or sexual abuse in childhood. Together, these findings reiterate that individuals with obesity experience considerably more traumatic experiences and current trauma symptoms than the general population, which may prove detrimental to their mental health and weight management.

### Implications

Previous research on the psychological mechanisms of obesity has primarily concentrated on acute psychopathology. This study contributes to elucidating the relationship between obesity and more complex and entrenched psychological processes. Overall, our findings indicated that individuals with obesity endorsed significantly more maladaptive schemas and schema modes and significantly less healthy schema modes than individuals with normal weight. In addition, individuals with obesity reported significantly more childhood trauma as well as significantly more PTSD symptoms within the last month than normal weight individuals. These findings have several clinical implications for the management of obesity and mental health more broadly.

First, they highlight the importance of comprehensively assessing maladaptive schemas and schema modes in weight intervention programs. Identifying how patients view themselves, others, and the world, and their primary coping strategies, may provide valuable insight for targeted intervention. For instance, our findings revealed that individuals with obesity principally display a psychologically withdrawn and submissive or passive coping style. This suggests that patients have developed ways to emotionally detach from their experiences, but do not employ effective strategies to manage their emotional difficulties. These maladaptive coping mechanisms may be activated by underlying schemas and schema modes that are entrenched within the patient (e.g., Punitive Parent, Vulnerable Child). This formulation is conceptually consistent with the schema mode model of obesity proposed by Basile et al. [16], which theorises that the Compliant Surrender and Detached Protector/Detached Self-Soother modes arise from the patient’s attempt to cope with the Punitive Parent and Impulsive Child modes. Importantly, this pattern of functioning may interfere with weight management over time. Therefore, identifying entrenched schemas and schema modes, including their origins and triggers, could be a critical first step to improving long-term weight management for individuals with obesity.

Second, as part of weight intervention, maladaptive schemas and schema modes should be addressed through psychological treatments such as schema therapy. Schema therapy integrates cognitive, behavioural, and experiential techniques to modify maladaptive coping strategies, reduce the influence of the internalised parent modes, satisfy unmet core emotional needs, and foster healthy functioning [7, 8]. Importantly, schema therapy focuses on establishing a safe therapeutic relationship that acknowledges the previously functional role of current maladaptive coping methods (e.g., in response to trauma) and substitutes them with more helpful coping strategies. If untreated, however, underlying schemas and schema modes may interfere with longer-term outcomes due to their pervasive and enduring nature. Therefore, intervention for obesity must inevitably replace maladaptive coping methods (e.g., binge eating) with more functional and adaptive responses. By developing more adaptive coping strategies and minimising maladaptive schema modes, individuals with obesity could remove barriers that potentially impede diet and physical activity and improve longer-term weight management.

Third, these findings emphasise the importance of comprehensively assessing trauma history in weight intervention programs. Research indicates that individuals with obesity with a history of childhood abuse and PTSD demonstrate significantly more eating psychopathology, physical health concerns, and psychological difficulties (e.g., substance misuse, body image) than those without a history of trauma or PTSD [13]. Further, childhood and adulthood trauma has been linked to eating disorders such as BED [12, 14]. If untreated, BED may hinder weight loss outcomes and contribute to weight regain for those undertaking bariatric surgery [39, 40]. Therefore, identifying individuals with a history of trauma may be critical to detecting those vulnerable to adverse medical and psychological outcomes. Importantly, however, individuals with obesity may underreport their clinical symptoms to appear more favourable during psychological assessments [41]. Similarly, they may require intensive intervention over time to accurately recall their traumatic experiences. As a result, it is essential for all health professionals within multidisciplinary teams to be cognisant of the markers of trauma to identify those at increased risk of less successful outcomes.

Finally, these findings reinforce the importance of treating mental health difficulties in weight intervention programs. Psychological difficulties (e.g., eating psychopathology, depression, anxiety, binge eating) have been linked to weight regain after bariatric surgery [5, 42] and may interfere with longer-term weight management. As evidenced in our study, individuals with obesity also experience more complex and enduring psychological difficulties, including increased childhood trauma, PTSD symptoms, and maladaptive schemas and schema modes. Childhood trauma may also represent a transdiagnostic risk factor for other mental health concerns, including depression and psychosis [43]. Future obesity treatment models could consider routine screening for childhood trauma, PTSD, and maladaptive coping strategies, to identify those requiring more intensive intervention. This will contribute to a more holistic and individualised conceptualisation of weight management and could attenuate the influence of psychological mechanisms on long-term weight maintenance.

### Limitations, Strengths, and Future Directions

Our results should be considered in the context of several limitations. First, we did not explicitly assess all types of childhood maltreatment (e.g., bullying, parental domestic violence, serious accident/death). As a result, some participants may have experienced childhood adversities that were not captured in this study. For example, MacDonald et al. [44] found that almost one-third of their participants experienced childhood adversities that were not identified by the CTQ-SF. In addition, childhood maltreatment is often assessed retrospectively, which may contribute to recall inaccuracies. These inaccuracies may be deliberate, due to fear of negative evaluation from others (e.g., stigma associated with the experience), or unintentional, because of an emotional avoidance of the traumatic experience. Future research could utilise longitudinal designs to minimise the potential effects of recall bias when assessing childhood trauma. Furthermore, as proposed by Pilkington et al. [9], future research could investigate the developmental period of maltreatment (e.g., first 12 months of life, adolescence) as well as the impact of different perpetrators (e.g., mother, father, grandparent, sibling, peer) on outcomes.

Second, responses on schema-related questionnaires may be influenced by active schemas or schema modes at the time of the assessment. For example, individuals in an avoidant coping mode (e.g., Detached Protector) may inadvertently minimise their clinical symptoms, whereas individuals in a Vulnerable Child mode may unintentionally inflate their concerns. Similarly, although EMS are considered relatively stable, it is unclear whether certain questions (e.g., trauma) prime responses or elicit emotional states that influence responding style. In addition, it is unclear whether certain psychiatric medication attenuates schema activation. The potential variability in schema activation at the time of the assessment may contribute to differences in outcomes across the literature, but future research is required to verify this claim. In addition, future research could examine these outcomes over multiple time points to circumvent potential response bias associated with active schemas and schemas modes.

Third, previous research has shown that treatment-seeking individuals with obesity may underreport their clinical symptoms due to fear of treatment ineligibility [41]. Supporting this claim, our findings indicated that 38.8% of participants in the obesity group provided scores that indicated possible underreporting of childhood maltreatment. This social desirability bias was also observed in the normal weight group, with 25% of participants possibly underreporting childhood maltreatment. Furthermore, we would expect that participants responded with a similar bias to the other questionnaires in our study; however, this was not formally assessed. Therefore, our results should be considered carefully, as they may underestimate the actual prevalence of childhood maltreatment in the current sample as well as the severity of EMS and schema modes. Future research could utilise a multi-method approach (e.g., self-report questionnaire, semi-structured interview) when assessing these constructs to mitigate potential social desirability bias.

Fourth, our sample predominantly consisted of female participants, with a large portion recruited from a clinical setting. Notably, this clinical setting resides in a lower socioeconomic area in Australia than the sample recruited by Anderson et al. [15] and is characterised by greater medical complexities than a community sample. Australian adults residing in lower socioeconomic regions have a greater likelihood of elevated psychological distress and adverse health complications such as obesity, relative to individuals from higher socioeconomic regions [45, 46]. Importantly, community participants were recruited from the same socioeconomic area in Australia to minimise sociodemographic variance across groups. In addition, we excluded participants with severe mental and physical illness and cognitive/intellectual impairment to circumvent the influence of more severe presentations on outcomes. Nonetheless, future research could replicate these findings in a community sample with similar sex distribution and socioeconomic factors, which may improve the external validity of these results. Future studies could also examine whether trauma exposure and coping responses vary among men and women, especially in the context of potential biological differences and culturally acceptable forms of coping. For example, previous research has found that men are more susceptible to environmental stressors (e.g., abuse, neglect) due to differences in neural development [47], which may have implications for longer-term outcomes. Moreover, future research could investigate the relationship between the onset of obesity (i.e., childhood, adolescence, adulthood) and childhood trauma, EMS, and schema modes.

Finally, there are several notable strengths in the current study. First, we utilised the YSQ-S3 and refined, 118-item SMI, which are the latest iterations of the schema questionnaires. This extends previous literature that compared EMS and schema modes in individuals with obesity and normal weight controls using the YSQ-S2 and 124-item Schema Mode Inventory, respectively [15–17]. Importantly, though, the motivation and time taken to complete these questionnaires may have contributed to respondent boredom or fatigue, possibly impacting recruitment and the completion rate. Second, our clinical sample comprised treatment-seeking individuals with obesity attending a tertiary weight intervention service, with all participants containing a BMI in the obesity range. This extends the findings of previous studies that combined overweight and obesity participants into one group [16]. The use of a combined overweight and obesity group may contribute to differences in outcomes, but future research is required to investigate this further. In addition, the inclusion of a treatment-seeking sample elaborates previous research that compared schema modes in normal weight controls and individuals with obesity from the community [16].

## Conclusion

This was the first study to utilise the YSQ-S3 and revised, 118-item, SMI to compare EMS and schema modes in normal weight controls and individuals with obesity attending a tertiary weight intervention service. This study also examined differences in childhood trauma and PTSD symptoms among treatment-seeking individuals with obesity and normal weight controls. Overall, results revealed that individuals with obesity endorsed significantly more maladaptive schemas and schema modes and significantly less healthy schema modes than individuals with normal weight. In addition, individuals with obesity reported significantly more childhood trauma as well as significantly more PTSD symptoms within the last month than normal weight individuals. Future obesity treatment models should incorporate routine assessment of these psychological difficulties, which may contribute to a more holistic conceptualisation of obesity. Moreover, addressing these underlying mental health concerns through evidence-based psychological interventions such as schema therapy, may attenuate the influence of psychological processes on weight management, and could improve the long-term success of patients with obesity.

## Supplementary Information

**Additional file 1:** 

**Additional file 2:** 

## Data Availability

The datasets used and/or analysed during the current study are available from the corresponding author on reasonable request.
