# Supplementary material for: Childhood trauma, posttraumatic stress disorder symptoms, early maladaptive schemas, and schema modes: a comparison of individuals with obesity and normal weight controls
Source: BMC Psychiatry. 2022 Jul 30;22:517. doi: 10.1186/s12888-022-04169-7 (PMC9339192; doi:10.1186/s12888-022-04169-7)
Supplement: Supplementary file 1 — Additional file 1: [file 12888_2022_4169_MOESM1_ESM.docx]

**Appendix A**

**Description of Early Maladaptive Schemas in the Young Schema Questionnaire, Short Form, Version 3**

| Early maladaptive schemas | Description |
| --- | --- |
| Disconnection and rejection domain | |
| Abandonment | The perception that one’s important relationships with others are unstable and will not last |
| Mistrust/abuse | The expectation that others will manipulate, humiliate, abuse, or treat one poorly |
| Emotional deprivation | The belief that others will not fulfil one’s emotional needs and that they will remain unsatisfied |
| Defectiveness/shame | The perception that one is inferior, defective, unlovable, or unwanted by others |
| Social isolation/alienation | The sense of not belonging to communities and social groups due to feeling dissimilar to others |
| Impaired autonomy and performance domain | |
| Dependence/incompetence | The belief that one is less capable of managing daily responsibilities without the aid of others |
| Vulnerability to harm and illness | The apprehension that catastrophic illness and injury is unpreventable and imminent |
| Enmeshment | Having an extreme emotional connection with significant others to the detriment of one’s individuality |
| Failure | The belief that one will inevitably fail and be less successful than peers in important areas of life |
| Impaired limits domain | |
| Entitlement/grandiosity | The belief that one is entitled, deserves special privileges, exempt to normal rules, and superior to others |
| Insufficient self-control | Difficulty exhibiting self-control, delaying gratification, or inhibiting extreme impulses and emotions |
| Appendix A (continued) | |
| Early maladaptive schemas | Description |
| Other-directedness domain | |
| Subjugation | Surrendering control to others due to the perception that one is being coerced |
| Self-sacrifice | Voluntarily prioritising the needs of others at the expense of one’s own needs and desires |
| Approval-seeking | Excessive effort on obtaining attention, recognition, or approval from others in lieu of developing the self |
| Overvigiliance and inhibition domain | |
| Negativity/pessimism | Preoccupation with problematic or negative aspects of life and a minimisation of the positive aspects |
| Emotional inhibition | Excessive inhibition of emotion due to the belief that emotions are unnecessary or unpleasant to display |
| Unrelenting standards | The belief that one must achieve extremely high standards of performance/behaviour to prevent criticism |
| Punitiveness | The perception that harsh punishment is warranted when people make mistakes |
