# Supplementary material for: Childhood trauma, posttraumatic stress disorder symptoms, early maladaptive schemas, and schema modes: a comparison of individuals with obesity and normal weight controls
Source: BMC Psychiatry. 2022 Jul 30;22:517. doi: 10.1186/s12888-022-04169-7 (PMC9339192; doi:10.1186/s12888-022-04169-7)
Supplement: Supplementary file 2 — Additional file 2: [file 12888_2022_4169_MOESM2_ESM.docx]

**Appendix B**

**Description of Schema Modes in the Revised, 118-Item, Short Schema Mode Inventory**

| Schema modes | Description |
| --- | --- |
| Child modes | |
| Vulnerable child | Feels abandoned, unloved, sad, worthless, isolated, rejected, lonely, unsupported, and frightened |
| Angry child | Experiences strong anger and frustration due to unmet physical or emotional core needs |
| Enraged child | Feels rage and acts with uncontrollable aggression and violence towards objects and people |
| Impulsive child | Acts on impulses or desires in uncontrolled or selfish ways irrespective of the implications |
| Undisciplined child | Experiences difficulties completing mundane or monotonous tasks and easily gives up or becomes frustrated |
| Happy child | Feels loved, spontaneous, connected, safe, understood, and has their core emotional needs satisfied |
| Coping modes | |
| Compliant surrender | Behaves in a passive, submissive, and compliant way; tolerates or accepts abuse and does not actively pursue healthy needs |
| Detached protector | Acts in an avoidant, emotionally withdrawn, and detached manner; disconnects from others and refuses to accept the help of others |
| Detached self-soother | Psychologically detaches from emotions by engaging in distracting, stimulating, or soothing activities |
| Self-aggrandiser | Acts in a grandiose, abusive, competitive, and demeaning way to overcompensate or inflate their sense of self |
| Bully/attack | Acts in an intimidating and aggressive way to protect oneself from actual/perceived threat or to obtain desires |
| Appendix B (continued) | |
| Schema modes | Description |
| Parent modes | |
| Punitive parent | Experiences a harsh, unforgiving, and critical internalised parent/caregiver voice, which punishes or blames the self for displaying or feeling normal needs that were similarly punished in childhood when expressed |
| Demanding parent | Constantly places excessive pressure on oneself to meet extremely high and internalised standards |
| Healthy adult mode | |
| Healthy adult | Practices appropriate adult functioning and engages in daily adult responsibilities and pleasurable practices in a functional way |
